# Beyond Minoxidil: Off-Label Therapies for Male Androgenetic Alopecia—A Systematic Review with Network Meta-Analyses

**DOI:** 10.3390/medicina62071282

**Published:** 2026-07-03

**Authors:** Aditya K. Gupta, Shannon A. H. Compton, Amanda Liddy, Mesbah Talukder, Tong Wang, Mary A. Bamimore

**Affiliations:** 1 Division of Dermatology, Temerty Faculty of Medicine, University of Toronto, Toronto, ON M5S 1A8, Canada; 2 Mediprobe Research Inc., London, ON N5X 2P1, Canada; scompton@mediproberesearch.com (S.A.H.C.); aliddy@mediproberesearch.com (A.L.); mtalukder@mediproberesearch.com (M.T.); twang@mediproberesearch.com (T.W.); mbamimore@mediproberesearch.com (M.A.B.); 3 School of Pharmacy, BRAC University, Dhaka 1212, Bangladesh

**Keywords:** androgenetic alopecia, off-label therapies, topical minoxidil

## Abstract

*Background*: There is limited evidence on comparative effectiveness of off-label therapies approved by the United States Food and Drug Administration (FDA) for androgenetic alopecia (AGA) and the unregulated ones. *Objective*: To evaluate and compare the molecular mechanisms, clinical efficacy, and safety of off-label therapies for male AGA through a network meta-analysis (NMA). *Methods*: A systematic literature search in PubMed, Scopus, Web of Science, and ClinicalTrials.gov was conducted on 23 January 2026. We examined studies that assessed mean 6-month change in total hair density of off-label monotherapies, including topical minoxidil (2%, 5%) for male AGA. Sensitivity analyses were conducted. *Results*: Our search identified 23 studies (15 off-label and 8 topical minoxidil). We found topical minoxidil 5% to be ranked most effective. Other effective therapies included melatonin (topical), diaminopyrimidine oxide 1% (topical), procyanidin 1% (topical), saw palmetto (topical), exosome-based formulation containing plant-derived extracts (subcutaneous), cetirizine 1% (topical) and finasteride (topical). *Conclusions*: Our results substantiate topical minoxidil 5% being the most effective option; our findings also suggest that other off-label therapies demonstrate potential clinical benefit, warranting further high-quality comparative trials.

## 1. Introduction

Androgenetic alopecia (AGA) is the most predominant type of hair loss in men, affecting up to 80% of men by age 70 years [1]. At the molecular level, AGA is a multifactorial process largely explained by progressive hair follicle miniaturization and a shorter anagen (growth) phase. These changes are driven by dihydrotestosterone (DHT)-mediated androgen receptor signaling within the dermal papilla cells. Elevated local DHT levels, increased activity of the enzyme 5α-reductase (which converts testosterone into DHT), and subsequent downstream dysregulation of growth and inhibitory signaling pathways collectively contribute to follicular regression and impaired hair cycling [2].

Despite the high prevalence of AGA, therapeutic options approved by the United States (U.S.) Food and Drug Administration (FDA) for this condition are mainly limited to two agents: oral finasteride 1 milligram (mg) and topical minoxidil (2% and 5%). Oral finasteride, a prescription medication, inhibits type II 5α-reductase activity, thereby reducing DHT levels; topical finasteride is designed to reduce systemic exposure compared with oral finasteride, although systemic absorption and DHT suppression may still occur [2]. In contrast, topical minoxidil (2% or 5%) is the only U.S. FDA-approved over-the-counter treatment for AGA, making it readily accessible and commonly used as an initial therapeutic option. Although its mechanisms are not fully understood, minoxidil may promote hair growth via potassium channel activation, Wnt/β-catenin signaling, anti-inflammatory effects, and possible antiandrogen activity [3]. However, individual variability in treatment response and side effects, such as contact dermatitis, hypertrichosis, and pruritus, highlights the need for additional therapeutic options [3].

Growing evidence suggests that alternative off-label therapies—such as natural compounds and therapies not currently indicated for AGA by the U.S. FDA—may be effective in attenuating male AGA. These alternatives modulate molecular pathways implicated in AGA, including androgen metabolism, dermal papilla and follicular activation, inflammatory and oxidative modulation, and growth factor signaling. As such, they may provide alternative or adjunctive strategies for individuals unwilling or unable to use conventional therapies.

We previously evaluated conventional and non-conventional over-the-counter (OTC) therapies for male androgenetic alopecia (AGA). The present study extends our previous work by focusing specifically on off-label therapies and substantially expanding the evidence base. In this review, we emphasize molecular mechanisms, clinical efficacy, and safety. We included randomized clinical trials, comparative clinical studies, and observational studies evaluating off-label treatments and topical minoxidil (2% and 5%) in adults with AGA, with 6-month change in total hair density as the primary outcome.

## 2. Materials and Methods

We conducted a systematic literature search in PubMed, Scopus, Web of Science, and ClinicalTrials.gov on 23 January 2026, using keywords and Medical Subject Heading (MeSH) terms for ‘androgenetic alopecia’, ‘patterned baldness’, and ‘hair loss’; we also used terms pertaining to commonly studied off-label or adjunctive therapies (e.g., saw palmetto/Serenoa repens, procyanidin, cetirizine, rosemary oil, watercress, marine complex, marine extract, melatonin, botulinum toxin/botox, Kopexil, topical finasteride, and exosome). For example, a PubMed search included terms such as: ((androgenetic alopecia*[Title/Abstract] OR male AGA*[Title/Abstract] OR male pattern hair loss*[Title/Abstract]) AND (saw palmetto*[Title/Abstract] OR Serenoa repens*[Title/Abstract])). The conduct and reporting of this study followed the PRISMA guidelines for NMAs, and the review protocol was registered on the Open Science Framework (OSF) to enhance transparency and reproducibility (registration link: https://doi.org/10.17605/OSF.IO/R4XZC). Details of search queries for the other databases are provided in Table S1 of the Supplement.

After search queries were established, two authors (MAB and MT) independently screened the deduplicated searches. The screening of titles, abstracts, and full texts was performed using eligibility criteria based on the patient, intervention, comparator, and outcome (PICO) framework. Eligible studies were interventional studies that studied male patients with AGA (P), examined off-label monotherapies including topical minoxidil (2% or 5%) (I), also examined inactive controls (placebo or vehicle) or other active agents as comparators (C), and reported 6-month change in hair density (hairs/cm^2^) (O). Data extraction and risk-of-bias (RoB) assessment were also performed independently by the same reviewers (MAB and MT). Any discrepancies between the two authors were resolved through discussion with a third author (AKG). We used the Cochrane Collaboration’s RoB tools, such as the traffic plot, to present our qualitative summary for study-level evaluation of evidence quality. Studies were excluded if they included women (without analyses being separated by sex), were not limited to AGA, had less than a 24 week of follow-up, or did not report sufficient quantitative outcome data. We only included evidence in the English language. Screenings were performed with Rayyan software.

The extracted data, which were organized into spreadsheets, were used to conduct an NMA that attempted to quantitatively determine the relative effectiveness of the therapies we examined insofar as the 6-month change in total hair density (in hairs/cm^2^). The extracted data pertained to intervention details, reference (i.e., year of publication and first authors’ last name), patients’ demographics (i.e., age and disease severity [as per the Norwood–Hamilton classification]), and outcome information (i.e., mean, standard deviation [SD] and sample size).

Furthermore, we conducted a node-splitting analysis of inconsistency to statistically determine the level of agreement between direct and indirect evidence.

Our quantitative analyses included data from randomized and observational evidence (i.e., single-arm studies); hence, our NMA combined both randomized and non-randomized data, a form of knowledge synthesis that is not uncommon. Single-arm studies were incorporated into a connected network of randomized trials by recommendations from the peer-reviewed literature [4,5]: the single-arm trials were matched to a study whose distribution of potential effect modifiers (i.e., age and disease severity) resembled the single-arm study the most. The matched study was treated as an additional arm in the study that the single-arm study was matched to; thereafter, outcome data were combined by naïve pooling—where there is no demarcation of study design. The naïve pooling approach is widespread throughout the literature. Because of our incorporation of observational evidence, we conducted sensitivity analyses, where non-randomized evidence was excluded. For our base model, we ran a Bayesian fixed-effect NMA with weakly informative priors. For all NMAs, we used the multinma package in R version 4.3.2 (R Foundation for Statistical Computing, Vienna, Austria) in RStudio Desktop (Posit Software, PBC, Boston, MA, USA), and the key outcome variable was the per arm mean 6-month change in total hair density and the standard deviation of this mean; wherever papers did not directly provide these, the literature guided our calculations for their estimation [6,7].

An NMA estimates each intervention’s surface under the cumulative ranking curve (SUCRA) value as per the following formula:

$${\mathrm{SUCRA}}_{k}=\frac{\sum_{c=1}^{b-1}{Cum}_{k,c}}{b-1}$$

where *k*, *c*, and *b* represent an intervention of interest, an intervention’s rank probability and total number of interventions in the network, respectively. The *Cum_k,c_* denotes the cumulative probability that intervention k is among the top c treatments. An intervention’s SUCRA (i.e., SUCRA*_k_*), whose value is between 0% and 100% (inclusive), corresponds to their overall rank for efficacy—where the most efficacious intervention is represented by the highest SUCRA value. In addition to SUCRA values, an NMA also generates relative effects (RE) for every possible pairwise comparison of interventions. For our study, the point estimate was mean difference (MD) of the mean 6-month change. A 95% credible interval (CrI) was estimated for every MD. All REs for the base NMA were presented in league table.

We depicted the geometry of our network with network plot, a graph of nodes and edges; a node corresponds to the vertices while the line between two vertices is referred to as the edge. The edge represents the comparison of two interventions whose efficacy was compared in an actual head-to-head trial [8].

We conducted model diagnostics—for base and sensitivity models. Model convergence was assessed using the Gelman–Rubin potential scale reduction factor (i.e., R-hat). Convergence was considered adequate when R-hat values approached 1.00. Model fit was evaluated using the deviance information criterion (DIC). For random-effects models, between-study heterogeneity was assessed using the heterogeneity parameter tau (τ). Node-splitting analyses were conducted with gemtc package in R. All analyses were performed with R version 4.3.2.

## 3. Results

**Figure 1:**
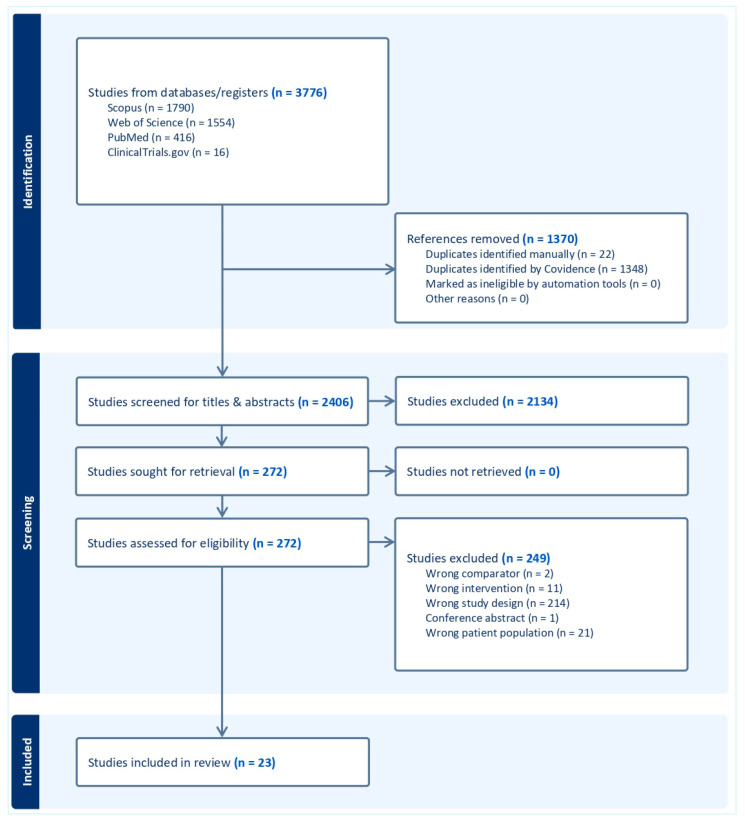
Schematic for the identification of eligible studies.

**Figure 2:**
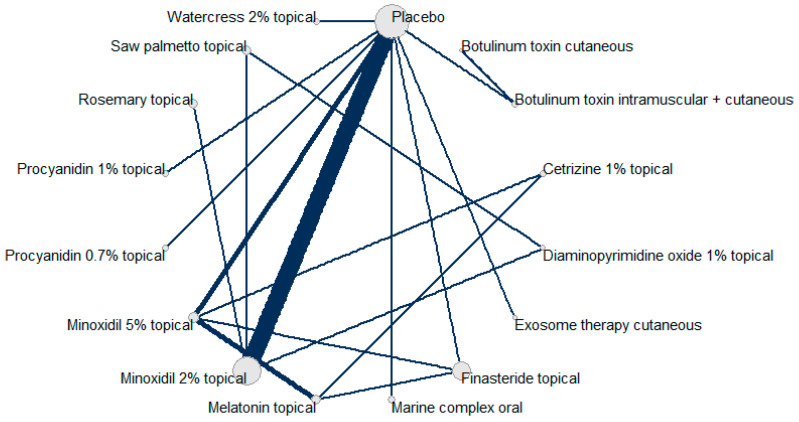
Network plot of interventions for male androgenetic alopecia included in the network meta-analysis (base model). Nodes represent interventions (15 active comparators and pooled control), and edges indicate direct comparisons reported in clinical trials [9,10,11,12,13,14,15,16,17,18,19,20,21,22,23,24,25,26,27,28,29,30,31]. Lima-Galindo et al. (2025) [12] and Melo et al. (2024) [11] evaluated botulinum toxin using different administration routes (the intramuscular and subcutaneous (IM + SC) vs. intramuscular and intradermal (IM + ID)). For network consistency, intradermal and subcutaneous routes were grouped as “cutaneous.” The nodes are proportional to the sample size, and the edges are proportional to the number of studies.

Following screening and eligibility assessment, 15 trials for off-label interventions were identified, some of which were randomized trials that had a topical minoxidil arm (Table 1). The systematic literature search and study selection process are summarized in the Preferred Reporting Items for Systematic Reviews and Meta-Analyses (PRISMA) flow diagram (Figure 1). Furthermore, Table 1 presents non-minoxidil therapies at the agent level; it does not, for example, distinguish between procyanidin 1% and 7%. The same literature search identified eight trials on topical minoxidil (two of which pertained to minoxidil 5%—and the remaining six to minoxidil 2%). Hence, our review—and consequently our NMAs—was based on a total of 23 studies; the network plot for our base model is shown in Figure 2 [9,10,11,12,13,14,15,16,17,18,19,20,21,22,23,24,25,26,27,28,29,30,31]. A summary of included studies’ characteristics is detailed in Table 2 [9,10,11,12,13,14,15,16,17,18,19,20,21,22,23,24,25,26,27,28,29,30,31]. A qualitative summary of included studies’ RoB are presented in Figure S1 of the Supplement.

For network construction, placebo and vehicle comparators were combined into a single control node that served as the common reference comparator across studies. This approach facilitated a connected evidence network. Hence, the resulting network consisted of active treatment nodes and an ‘amalgamated’ control node (Figure 2; Table 2). An exception was made to include the study by Amini et al. (2025) [23], as it was the only suitable study that investigated an exosome-based therapy on male AGA; the authors examined the impact of exosome-based formulation containing plant-derived extracts at 16 weeks.

To produce ‘analysis-ready’ data for our NMAs, we performed various estimations to obtain the mean 6-month change in hair density (in hairs/cm^2^) and the ± standard deviation (SD) of this mean. Some of the included studies directly provided these two values directly, while some did not (Table 2). In Table 2, we reported total hair density at baseline as described in respective papers. For instance, Wessagowit et al. (2016) [22] reported a hair count of 1736.369 per 2.54 cm^2^—and this is what we directly presented in Table 2. When we produced the analysis-ready estimates, we conducted calculations that were guided by the literature—as mentioned earlier. A detailed calculation guide is provided in Table S2 in the Supplement. Only one included study utilized a split-scalp design, and no adjustment for split-scalp was undertaken as it was deemed not to substantially influence inferences.

We assessed the distribution of key potential effect modifiers, including age, baseline disease severity, treatment duration, study design, and outcome measurement methods. While some variation was observed across studies, there were no obvious imbalances suggestive of a violation of the transitivity assumption. However, given the incomplete reporting of study characteristics and the limited number of studies available for several interventions, the possibility of residual intransitivity cannot be entirely excluded (Table 2).

To assess the robustness of the network meta-analysis findings, four sensitivity analyses were conducted.

• First, all single-arm studies were excluded, and the network was restricted to randomized controlled trials (RCTs) using a fixed-effects model.
• Second, the same RCT-only network was analyzed using a random-effects model.
• Third, a fixed-effects RCT-only network was constructed after excluding Amini et al. (2025) [23], which evaluated an exosome-containing plant extract formulation and was included in the primary analysis despite a shorter follow-up duration than the prespecified eligibility criterion.
• Fourth, a random-effects version of this restricted network was evaluated.

**Figure 3:**
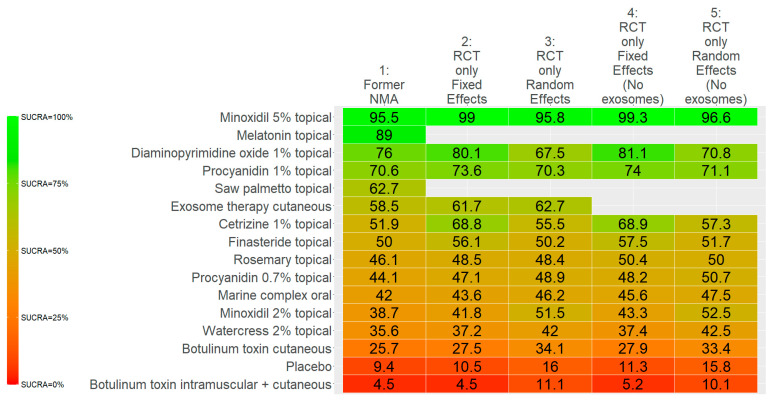
**Kilim plot comparing SUCRA values across the primary and sensitivity network meta-analysis models.** Treatments are displayed on the *y*-axis and network meta-analysis models on the *x*-axis. Numerical values within cells represent the surface under the cumulative ranking curve (SUCRA), while cell colors correspond to SUCRA values on a continuous gradient ranging from 0% (with 0% being most red) to 100% (with 100% being most green). Left to right: Model 1 represents the primary network meta-analysis including all eligible studies (fixed-effects model). Model 2 represents the randomized controlled trial (RCT)-only fixed-effects network. Model 3 represents the RCT-only random-effects network. Model 4 represents the RCT-only fixed-effects network excluding Amini et al. (2025) [23], which evaluated an exosome-containing plant extract formulation. Model 5 represents the corresponding RCT-only random-effects network excluding Amini et al. (2025) [23]. The substantial congruence in color gradients and SUCRA values across models indicates that treatment rankings were generally robust to the exclusion of single-arm studies, exclusion of Amini et al. (2025) [23], and alternative modeling assumptions [9,10,11,12,13,14,15,16,17,18,19,20,21,22,23,24,25,26,27,28,29,30,31].

These sensitivity analyses were undertaken to assess the influence of non-comparative evidence, model specification, and inclusion of the Amini et al [23]. study on treatment rankings and relative treatment effects. To facilitate comparison across analyses, treatment rankings from the primary network and all sensitivity analyses were summarized using a kilim plot. Detailed explanation of a kilim plot is detailed in the Supplement (Table S3). The kilim plot in Figure 3 presents comparators’ SUCRA values from base NMA and sensitivity analyses. Comparators’ pairwise relative effects from the base model are presented in Figure S2 the Supplement. Kilim plot showed high congruence in comparators’ SUCRA ranks across base and sensitivity models.

For each model, statistical consistency was evaluated using node-splitting analyses for inconsistency—and the results thereof are provided in the Supplement (Table S4); across the base and sensitivity models, there was no evidence of statistical inconsistency.

Topical 5% minoxidil was more effective than cetirizine 1% (mean difference [MD] = 13.83 hairs/cm^2^, 95% credible interval [CrI] = [4.05–23.64] hairs/cm^2^) and rosemary oil (MD = 15.17 hairs/cm^2^, 95% CrI = [2.87–27.39] hairs/cm^2^) (Figure S2, Supplement).

Across the base NMA and all four sensitivity analyses, convergence diagnostics indicated satisfactory model performance because the R-hat value of parameters was 1.00. Model diagnostics are presented in Table S5 of the Supplement.

We undertook a separate, in-depth review of each off-label therapy to further assess their individual efficacy, safety, and potential clinical utility. Table 3 outlines the mechanism of action for each of the therapies outlined in this study.

### 3.1. Finasteride (Topical)

**Figure 4:**
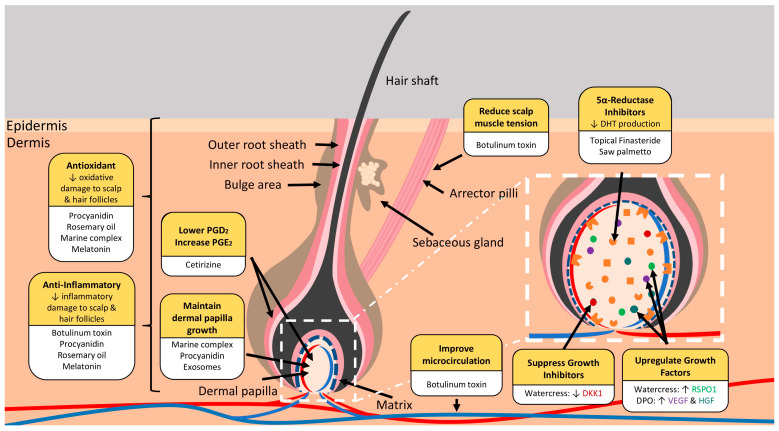
Molecular mechanisms of action of off-label AGA therapies [2,13,17,18,20,33,34,35,36,37,38]. DPO = diaminopyrimidine oxide; PGD_2_ = prostaglandin D_2_; PGE_2_ = prostaglandin E_2_; DKK1 = Dickkopf-1; RSPO1 = R-spondin 1; VEGF = vascular endothelial growth factor; HGF = hepatocyte growth factor (HGF). ↑ indicates an increase, and ↓ indicates a decrease.

Topical finasteride is a type II 5α-reductase inhibitor that blocks the conversion of testosterone to DHT. By reducing local DHT concentrations, finasteride limits androgen receptor activation in dermal papilla cells, attenuating follicular miniaturization and promoting terminal hair growth (Figure 4) [33]. Unlike oral finasteride, which can cause systemic androgen suppression and sexual adverse effects, the topical formulation minimizes these risks by acting locally on the scalp [2].

Similarly, subcutaneous long-acting finasteride injections are under investigation; a phase 1 open-label study suggests that 12, 24, and 36 mg injections are well-tolerated and achieve plasma concentrations comparable to daily oral dosing, despite being administered once every four weeks [39].

#### 3.1.1. Clinical Evidence

Few RCTs have evaluated topical finasteride in men with AGA (Table 2). In a Randomized, placebo-controlled, three-arm phase III trial with oral finasteride as an active reference comparator, Piraccini et al [9]. reported that a topical finasteride 0.25% solution significantly increased total hair count on the vertex compared with placebo, with a mean change in hair count of 20.2 hairs/cm^2^ and 6.7 hairs/cm^2^ from baseline to 24 weeks, respectively (*p* < 0.001). Topical finasteride had comparable efficacy to oral finasteride (20.2 vs. 21.1 hairs/cm^2^). These findings were consistent in a post hoc sensitivity analysis, which similarly showed a statistically significant advantage over placebo (16.3 vs. 6.3 hairs; *p* = 0.012) and comparable efficacy to the oral formulation [9].

Rossi et al. [10] compared the effectiveness of topical finasteride 0.25%, topical minoxidil 5%, and their combination in treating male AGA. The mean hair density increased from baseline to 6 months for all treatment groups; however, only the combination topical finasteride and topical minoxidil group reached significance between baseline and 6 months, with baseline hair density increasing from 101.9 ± 13.4 (mean ± standard error) to 183.5 ± 15.3 (mean ± standard error) hair/cm^2^ (*p* < 0.001). The increase in hair density from baseline to 6 months was significantly different between the combination treatment group and topical finasteride alone (*p* < 0.01) [10].

#### 3.1.2. Safety

Topical finasteride was generally well-tolerated across studies, with no serious adverse events reported. Mild adverse effects were infrequent. In one study, finasteride 0.25% led to discontinuation in 4 out of 189 patients, with mild erythema and pruritus also reported in 4 patients each [9]. In the other RCT, no adverse events were observed among the 23 patients treated with topical finasteride [10].

### 3.2. Botulinum Toxin (Intramuscular/Subcutaneous Injection)

Botulinum toxin is a neurotoxin that inhibits acetylcholine release at neuromuscular junctions, reducing muscle contraction. In AGA, this may reduce scalp muscle tension, improve microcirculation, and modulate anti-inflammatory signaling, creating a more favorable environment for hair follicle growth (Figure 4; Table 3) [34].

#### 3.2.1. Clinical Evidence

Botulinum toxin A (BTA) has been investigated in two RCTs for the treatment of male AGA (Table 2). In a 6-month triple-blind, split-scalp RCT, in which participants received BTA on one side of the scalp and saline on the other, Melo et al. reported that BTA treatment produced no significant improvement in hair density from baseline or in comparison with placebo on the frontal scalp or vertex (*p* > 0.3) [11].

Likewise, Lima-Galindo et al. reported that subcutaneous BTA injections, whether alone or combined with intradermal administration, produced no significant changes in hair density in frontal or occipital areas after 6 months compared to baseline, and no differences between groups (all *p* > 0.5) [12].

#### 3.2.2. Safety

Botulinum toxin was generally well tolerated across studies. One study reported no treatment-related adverse events [11]. In contrast, the second study found that 61.5% of participants experienced mild-to-moderate injection-site pain; however, there was no statistically significant difference in adverse events between the two groups [12].

### 3.3. Procyanidin (Topical)

Procyanidins are flavonoid antioxidants that may promote hair growth by mitigating oxidative stress and suppressing inflammatory signaling in the scalp. In addition, procyanidins may protect hair epithelial cells from TGF-β-induced apoptosis, which triggers the catagen (regression) phase, in part by activating the MAPK/MEK signaling cascade to stimulate cell proliferation and follicular viability [13,35] (Figure 4; Table 3).

#### 3.3.1. Clinical Evidence

Kamimura et al. [13] investigated the efficacy of topical procyanidin B2 1% in men with AGA (Table 2). Twice-daily application of procyanidin B2 led to a significant increase in vertex hair density from baseline after 6 months of treatment (*p* < 0.001). This improvement was significantly greater than that observed in the placebo group at 6 months (26.72 ± 22.12 vs. 0.32 ± 18.24 hairs/cm^2^; *p* < 0.005) [13].

Likewise, Takahashi et al. [14] evaluated topical procyanidin B2 0.7% in a separate 6-month RCT (Table 2). The treatment group again showed a significant increase in hair density compared to placebo after 6 months (*p* < 0.001). Patients who continued therapy for an additional 6 months experienced further gains, with an average regrowth of 23 hairs/cm^2^ at 12 months [14].

#### 3.3.2. Safety

In both trials, topical procyanidin B2 was well-tolerated. No treatment-related adverse events were observed during dermatological assessments, and no patients reported adverse effects, including with up to 12 months of continuous use [13,14].

### 3.4. Rosemary Oil (Topical)

Rosemary oil contains bioactive compounds such as rosmarinic and caffeic acid, which exhibit antioxidant, anti-inflammatory, and antimicrobial activity. These effects may support hair growth by improving scalp microcirculation and mitigating local oxidative stress and inflammation [33].

#### 3.4.1. Clinical Evidence

Panahi et al. [15] conducted a 6-month single-blind RCT that compared topical rosemary oil with a topical minoxidil 2%, a standard treatment in male AGA (Table 2). After twice-daily application, both groups showed a significant increase in hair count from baseline to 6 months (6.8 ± 31.73 and 2.3 ± 24.20 hairs/cm^2^, respectively; *p* < 0.05), with no significant differences between them, suggesting rosemary oil may be similarly effective to minoxidil 2% [15].

#### 3.4.2. Safety

Both topical rosemary oil and 2% minoxidil were linked to more frequent scalp itching after 6 months compared with baseline (*p* < 0.05). Despite this, scalp itching was more frequently reported in the minoxidil group, which also showed significantly higher rates of itching than the rosemary oil group at the same time point. although this side effect was reported more often in the minoxidil group (*p* < 0.05). No other adverse events were reported [15].

### 3.5. Marine Complex (Oral)

Marine complex supplements (e.g., Viviscal^®^) are derived from extracellular matrix proteins and glycosaminoglycans extracted from sharks and mollusks [16]. These supplements are proposed to promote hair growth by reducing oxidative stress, stimulating the proliferation of dermal papilla cells, and increasing alkaline phosphatase activity (a marker or follicular growth), thereby supporting follicular regeneration [36].

#### 3.5.1. Clinical Evidence

Albon conducted a 6-month double-blind RCT that evaluated oral marine complex supplementation in men with AGA (Table 2). Twice-daily supplementation significantly increased total and terminal hair density at the midline scalp compared with placebo at 6 months (both *p* = 0.001) [16].

#### 3.5.2. Safety

Oral marine complex supplementation was well-tolerated, with no adverse events reported [16]. Use is contraindicated in individuals with shellfish allergies due to the product’s shark and mollusk-derived components [16].

### 3.6. Cetirizine (Topical)

Cetirizine is a second-generation antihistamine that has been investigated for the topical treatment of AGA. It may inhibit prostaglandin D_2_ (PGD_2_), a negative regulator of hair growth that promotes the transition of follicles into the catagen (resting) phase and is elevated in AGA, thereby shifting prostaglandin signaling toward the release of prostaglandin E_2_ (PGE_2_), which promotes hair growth [17].

#### 3.6.1. Clinical Evidence

Mostafa et al [17]. conducted a 24-week RCT that compared a topical cetirizine 1% solution with topical minoxidil 5% in men with AGA (Table 2). Patients applied treatments twice daily for 16 weeks, followed by an 8-week treatment-free period. Both groups demonstrated significant increases in total hair density from baseline to week 24 (*p* < 0.05), however the minoxidil 5% group achieved greater improvement than the cetirizine 1% group (*p* < 0.05). Cetirizine was well tolerated compared with minoxidil [17].

#### 3.6.2. Safety

The cetirizine 1% topical solution was well tolerated, with no local or systemic side effects reported over the 24-week treatment period [17].

### 3.7. Watercress Extract (Topical)

Watercress extract, derived from *Nasturtium officinale,* contains bioactive compounds that modulate hair follicle activity. In dermal papilla cells, watercress extract upregulates R-spondin 1 (RSPO1), an agonist of the Wnt/β-catenin signaling pathway that stimulates follicular proliferation, and downregulates Dickkopf-1 (DKK1), a Wnt antagonist that suppresses hair follicle activity, thereby promoting hair growth [18].

#### 3.7.1. Clinical Evidence

In a 6-month double-blind RCT, Hashimoto et al. [18] assessed the efficacy of a topical watercress extract 2% solution in treating male AGA (Table 2). After twice-daily treatment, the watercress group showed significant increases in hair density (*p* < 0.05) and thickness (*p* < 0.01) at the vertex compared to placebo after 6 months [18].

#### 3.7.2. Safety

Topical watercress extract 2% was free of reported adverse effects throughout the 6-month trial [18].

### 3.8. Diaminopyrimidine Oxide (Topical)

Diaminopyrimidine oxide (Tradename = Kopexil), a synthetic analog of minoxidil, stimulates hair growth by upregulating hepatocyte growth factor (HGF), which promotes follicle proliferation and anagen induction. It also increases vascular endothelial growth factor (VEGF), which enhances follicular microcirculation by promoting angiogenesis, supporting hair follicle activity [37].

#### 3.8.1. Clinical Evidence

Amiri et al. [19] conducted a 24-week double-blind RCT comparing a niosomal diaminopyrimidine oxide 1% lotion and a niosomal minoxidil 2% lotion in the treatment of male AGA (Table 2). Hair density increased significantly from baseline to 6 months in both groups (*p* < 0.001), but diaminopyrimidine oxide produced nearly twice the improvement of minoxidil (57.6% vs. 25.6%, *p* < 0.001) despite the lower concentration [19].

#### 3.8.2. Safety

Topical diaminopyrimidine oxide 1% was well-tolerated over the 24-week trial period, with no reported side effects [19].

### 3.9. Melatonin (Topical)

Melatonin is a hormone that regulates the sleep–wake cycle and exhibits antioxidant, anti-inflammatory, and potentially anti-androgen properties. Although the exact mechanism remains unclear, these effects may help mitigate AGA by reducing oxidative stress and inflammation within hair follicles and by modulating androgen signaling, supporting hair growth [20].

#### 3.9.1. Clinical Evidence

Fisher et al. [20] conducted a 6-month open-label study of 0.0033% topical melatonin in men with early-stage AGA (Table 2). Nightly application significantly increased total hair count (by 29.2% at 3 months; 42.7% at 6 months) and hair density (by 29.1% at 3 months; 40.9% at 6 months) compared to baseline (both *p* < 0.001) [20].

Conversely, a 6-month open-label trial by Baldari et al. [21] evaluated nightly applications of 0.1 mg topical melatonin solution for 6 months on either the parietal or frontal regions of the scalp. Topical melatonin did not produce a statistically significant change in hair density when assessed across the full cohort or by scalp region. Nevertheless, a proportion of patients demonstrated a clinical response, with 51.7% showing improvement. In this subgroup, mean hair density increased significantly from 119.4 ± 40.1 to 156.3 ± 29.0 hairs/cm^2^ (*p* = 0.007). Overall, 18 patients (62%) experienced some degree of improvement [21].

#### 3.9.2. Safety

Topical melatonin was well-tolerated over 6 months. In both trials, one participant reported occasional mild pruritus at the application site (1/35 and 1/29, respectively). No other adverse events were reported [20,21].

### 3.10. Saw Palmetto Extract (Topical)

Saw palmetto (*Serenoa repens*), a small palm tree native to the West Indies and southeastern United States, produces lipid-rich berries that are used in extracts for the treatment of AGA. Saw palmetto extracts exert anti-androgenic effects, including the inhibition of type I and II 5α-reductase, interference with DHT–androgen receptor binding, and increased metabolism of DHT to androstenediol, which reduce DHT-mediated follicular miniaturization [33]. Only topical saw palmetto therapies are discussed in this review as studies involving oral formulations did not meet the inclusion criteria since they were only 4.6 months in duration [40] or did not report total hair density [41].

#### 3.10.1. Clinical Evidence

In a 24-week open-label cohort study, Wessagowit et al. [22] evaluated topical saw palmetto products for the treatment of AGA (Table 2). Patients applied a concentrated saw palmetto serum daily for 4 weeks and a saw palmetto lotion daily for all 24 weeks. Total and terminal hair counts at the vertex increased significantly from baseline to 24 weeks (both *p* < 0.001). Additionally, the authors reported that the median AGA stage improved from Norwood IV at baseline to Norwood III-v at week 24 (*p* < 0.0001).However, total hair count plateaued after week 12, suggesting discontinuation of the concentrated serum after week 4 may have limited benefits [22].

#### 3.10.2. Safety

Topical saw palmetto was associated with mild side effects in a trial of 49 patients, though none discontinued treatment. Reported effects included a cold sensation (16%) and mild burning (12%) at the application site, with less frequent reports of itchy scalp and forehead acne (~2% each) [22].

### 3.11. Exosomes

Exosomes are extracellular vesicles that mediate intercellular communication. In AGA, exosome therapies may stimulate dermal papilla cell activity and follicle cycling by upregulating the Wnt/β-catenin pathway and counteract DHT-mediated inhibition by suppressing the TGF-β1/SMAD pathway, together promoting hair regrowth [38]. Preliminary clinical evidence suggests exosome therapies can improve hair density and thickness [42]. They are typically delivered either by intradermal injections or topically after microneedling. Despite these promising results, exosome-based therapies remain experimental, and no exosome therapies are currently approved by the U.S. FDA [42].

#### 3.11.1. Clinical Evidence

In a 16-week RCT, Amini et al. [23] evaluated a subcutaneous exosome formulation containing *Ecklonia cava* and *Thuja orientalis* extracts in 20 men with AGA (Table 2). Participants were randomized to either receive injections of this exosome-based formulation containing plant-derived extracts or a 0.9% sodium chloride placebo for 4 bi-weekly sessions. The exosome-based group showed significant increases in total hair count over 16 weeks compared to baseline (*p* = 0.002) and compared to the placebo group (*p* = 0.006) [23].

#### 3.11.2. Safety

No significant adverse events were observed; however, 2 of the 10 patients in the treatment group experienced mild scalp irritation that resolved without intervention [23].

## 4. Discussion

The present study expands upon our previous network meta-analysis of conventional and non-conventional OTC treatments for male AGA in several important ways. First, the current network incorporates a larger and more diverse evidence base; the former examined 15 studies and nine active comparators while the current examined 23 studies and compared 15 active comparators. Second, in response to the methodological challenges inherent to this evidence base, we conducted multiple additional sensitivity analyses and expanded assessments of network assumptions, robustness, and consistency. Exclusion of all single-arm studies and restriction of the network to RCTs did not materially alter the overall pattern of treatment rankings. Similarly, treatment rankings remained broadly consistent when random-effects models were employed and when Amini et al. (2025) [23] was excluded from the network.

Visualization of SUCRA values using a kilim plot demonstrated substantial concordance in rank ordering across all five models, supporting the stability of the principal findings under alternative modeling assumptions and study inclusion criteria. Nevertheless, the incorporation of non-comparative evidence remains an important limitation. Single-arm and open-label studies are susceptible to placebo effects, regression to the mean, observer bias, and baseline imbalances that cannot be fully addressed in the absence of a concurrent control group. Consequently, therapies supported primarily by limited or non-comparative evidence should be interpreted with appropriate caution.

Several factors may explain the relative scarcity of large, blinded clinical trials evaluating off-label therapies for androgenetic alopecia. For instance, certain interventions, particularly injectable or procedural therapies, present practical challenges to blinding and placebo control.

Although several off-label therapies demonstrated promising efficacy signals, the quantity and quality of safety data varied considerably across studies. In addition to efficacy, the tolerability and safety profiles of these interventions should be considered when interpreting treatment rankings. Accordingly, clinicians should weigh potential benefits against the limited long-term safety evidence available for some interventions. Additional adequately powered randomized controlled trials with standardized outcome measures, longer follow-up periods, and rigorous reporting of adverse events are needed to better establish both efficacy and safety.

Topical minoxidil was intentionally included within our NMA study because it represents the most widely used and extensively studied topical treatment for this condition. As a result, minoxidil provides a clinically meaningful benchmark against which emerging and off-label therapies can be contextualized. The present review highlights that off-label therapies for male AGA support hair follicle vitality and regrowth through a number of different molecular mechanisms, some of which overlap with existing U.S. FDA-approved treatments.

Several off-label therapies share mechanisms with established treatments. For example, minoxidil, botulinum toxin, procyanidins, rosemary oil, and melatonin exhibit anti-inflammatory properties that may support follicular health and promote hair growth [3,13,20,33,34,35]. In parallel, topical finasteride and saw palmetto act as 5α-reductase inhibitors, targeting upstream androgen signaling in a manner similar to oral finasteride [2,33]. Other off-label agents provide novel mechanisms, with compounds like procyanidin, watercress extract, and diaminopyrimidine oxide enhancing downstream pro-anagen pathways (e.g., through MAPK/MEK, RSPO1, Wnt/β-catenin, HGF/VEGF) [13,18,35,37], supporting follicular proliferation. This mechanistic diversity underscores that male AGA is a multifactorial condition that can be targeted through multiple therapeutic strategies, highlighting the potential for alternative therapies for patients unresponsive to standard treatments, as well as combination approaches for patients seeking to optimize therapeutic efficacy.

In terms of clinical efficacy, topical minoxidil 5% demonstrated the greatest overall efficacy in improving hair density among the therapies reviewed. However, several off-label agents also showed clinically meaningful benefit, albeit to a lesser extent, including topical melatonin, topical diaminopyrimidine oxide 1%, procyanidin 1%, saw palmetto extract, topical exosome-based therapy, topical cetirizine 1%, and topical finasteride. Notably, safety profiles across trials suggest that these off-label treatments were well-tolerated, with predominately mild and transient adverse events and few discontinuations. The lack of significant adverse effects may improve adherence, particularly among patients reluctant to initiate medications which cause systemic androgen suppression, like oral finasteride, which can lead to undesirable side effects [2].

The evidence base for off-label therapies for AGA has expanded substantially in recent years. The inclusion of multiple randomized-controlled trials now permits more rigorous comparison among off-label treatments and U.S. FDA-approved therapies. Although topical minoxidil 5% remains the most effective treatment based on current data, several off-label agents demonstrated promising efficacy with favorable safety profiles, potentially offering alternative or adjunctive treatments to patients for whom standard therapies are ineffective, poorly tolerated, or undesirable. While further work is required to make more robust conclusions, existing data supports continued investigation through larger randomized trials with standardized endpoints, longer follow-ups, and directly controlled comparisons in order to clarify comparative effectiveness and optimize the management of male AGA.

### Limitations

This review has several limitations. Some of the off-label therapies were evaluated in a small number of trials. This review also focused exclusively on studies involving male patients, which limits the generalizability of our findings to women with AGA. However, this approach allowed us to reduce heterogeneity due to sex-related differences in hair loss patterns and treatment response, enabling more precise comparisons between men. Also, we only included AGA studies for which there were available quantitative data. We also cannot rule out the plausibility of publication bias. Despite these limitations, this review provides a rigorous synthesis of the available high-quality evidence on off-label therapies for male AGA.

## 5. Conclusions

Off-label therapies for male AGA act through diverse mechanisms, including antioxidant, anti-inflammatory, androgen-modulating, and anagen promoting effects. While topical minoxidil 5% remains the most effective treatment, several off-label agents show meaningful hair growth with favorable safety profiles, offering alternatives or adjuncts for patients unresponsive or resistant to standard therapies. Continued research with larger, controlled trials and standardized endpoints is needed to clarify comparative efficacy and guide optimized treatment strategies.

## 6. Future Directions

Although the U.S. FDA-approved therapies, topical minoxidil and oral finasteride, are effective for many men with AGA, limitations in efficacy, tolerability, and compliance have driven interest in alternative and adjunctive strategies. Emerging therapies target novel molecular mechanisms of action and drug delivery approaches. Whereas finasteride reduces DHT production via 5α-reductase inhibition, new topical agents directly target the androgen receptor through competitive androgen receptor antagonism (e.g., clascoterone [43]) and targeted androgen receptor degradation (e.g., GT20029 and AH-001) [44,45]. Other approaches explore beyond androgenic pathways, including prolactin receptor antagonism, which blocks prolactin receptor signaling to prevent follicle suppression and apoptosis (e.g., HMI-115, ABS-201 [46,47]) and mitochondrial pyruvate carrier inhibition, which promotes follicular stem cell proliferation via lactate-mediated integrated stress responses (e.g., PP-205 [48]). Improvements in drug delivery for existing therapies are also being explored to enhance adherence and ensure consistent follicular drug exposure, such extended-release oral minoxidil (e.g., VDPHLO1 [49]) or subcutaneous long-acting finasteride injections [39]. While long-term efficacy and safety data are limited, these approaches expand therapeutic options and may better address unmet clinical needs of men with AGA.

## Figures and Tables

**Table 1:** Overview of evidence quality for off-label AGA therapies in males.

Therapy (Agent-Level)	Total of Trials	Trial Type (n)			Total Patients (All Trials)	Randomized Trials (n)	Quality of Evidence ^1^
		Open-Label Trials	Placebo-Controlled Trials	Active Comparator Trials			
Topical finasteride	2	0	1	1	292	2	2
Botulinum toxin	2	0	1	1	28	2	2
Procyanidin	2	0	2	0	72	2	2
Rosemary oil	1	0	0	1	100	1	2
Marine complex	1	0	1	0	60	1	2
Cetirizine	1	0	1	0	30	1	2
Watercress extract	1	0	1	0	37	1	2
Diaminopyrimidine oxide	1	0	0	1	29	1	2
Melatonin	2	2	0	0	64	0	4
Saw palmetto	1	1	0	0	49	0	4
Exosomes	1	0	1	0	20	1	2

^1^ Quality defined by the Oxford Centre for Evidence Based Medicine. Level 1: systematic reviews. Level 2: individual randomized trials. Level 3: non-randomized controlled cohort studies. Level 4: Case series, case–control, or historically controlled studies. Level 5: mechanism-based reasoning [32]. The first column presents therapy at the agent level. For example, procyanidin pertains to the 7% and 1% formulations. Exomes pertain to exosome-based formulation containing plant-derived extracts.

**Table 2:** Study-level characteristics of studies included in our network meta-analysis.

Author	Agent	AGA Severity (Norwood–Hamilton)	Age, Range/Mean, Years (SD) ^A^	Sample Size	Design	Follow Up	Total Hair Density at Baseline (SD)	Mean Hair Density Change after 6 Months, Hair/cm^2^ (SD) ^B^
Piraccini 2022 [9]	Topical Finasteride 0.25%	III-v = 50 IV = 27 V = 28	32.5 (5.4)	105	randomized; whole head	24 weeks	change presented in graph	20.2 (29.7)
	Placebo	III-v = 45 IV = 31 V = 20	31.8 (4.9)	97	randomized; whole head	24 weeks	change presented in graph	6.7 (29.55)
	Oral Finasteride	III-v = 26 IV = 14 V = 8	32.3 (5.5)	48	randomized; whole head	24 weeks	change presented in graph	21.1 (39.96)
Rossi 2024 [10]	Topical Finasteride 0.25%	I = 3 II = 5 III = 4	23.7 (2.2)	12	randomized; whole head	6 months	136 (48)	18.00 (58.03)
	Topical Minoxidil 5%	I = 4 II = 6 III = 1	23.5 (2.2)	11	randomized; whole head	6 months	185 (55.4)	38.7 (61.19)
	Combination topical finasteride 0.25% and topical minoxidil 5%	I = 0 II = 11 III = 4 IV = 1 V = 3	25.3 (2.6)	19	randomized; whole head	6 months	101.9 (58.2)	81.6 (62.98)
Melo 2024 [11]	Botulinum toxin (Intramuscular + Intradermal)	II = 1 III = 6 IV = 3 V = 3	37.2 (5.8)	13	randomized; split-scalp	24 weeks	Frontal: 216 (56.4) Vertex: 216.9 (40)	Frontal: 2.7 (17.3) Vertex: 0.9 (13.4)
	Placebo		37.2 (5.8)	13	randomized; split-scalp	24 weeks	Frontal: 216.3 (48) Vertex: 217.5 (40.7)	Frontal: 3.0 (14.4) Vertex: 7.9 (13.1)
Lima-Galindo 2025 [12]	Botulinum toxin (Intramuscular + subcutaneous injections)	III = 1 III-V = 1 IV = 5 V = 1	42.5 (IQR: 27.5)	8	randomized; whole head	6 months	Frontal area (Median, IQR): 173.3 (54.25) Occipital area (Median, IQR): 130.05 (41.5)	Median (IQR): Frontal: −16.05 (64.2) Occipital: 1.15 (14.35)
	Botulinum toxin (Subcutaneous injections)	III = 1 III-v = 1 IV = 5 V = 0	41 (IQR: 28)	7	randomized; whole head	6 months	Frontal area (Median, IQR): 149.5 (68.6) Occipital area (Median, IQR): 142.8 (80.8)	Median (IQR): Frontal: 31 (87.5); Occipital: 21.1 (59.8)
Kamimura 2000 [13]	Topical Procyanidin 1%	I = 11 IV = 8	45	19	randomized; whole head	6 months	information presented graphically	26.72 (22.12)
	Placebo	I = 6, IV = 4	48	10	randomized; whole head	6 months	information presented graphically	0.32 (18.24)
Takahashi 2005 [14]	Topical Procyanidin 0.7%	I = 2 II = 8 IV = 11 ^C^	27–58 years	21	randomized; whole head	6 months	only mean change reported	6.6 (26.00)
	Placebo	I = 2 II = 11 IV = 9 ^C^	27–58 years	22	randomized; whole head	6 months	only mean change reported	−7.2 (16.20)
Panahi 2015 [15]	Topical Rosemary Oil	II = 37 III = 13 IV = 0	24.78 (3.67)	50	randomized; whole head	6 months	122.8 (48.9)	6.8 (31.73)
	Topical Minoxidil 2%	II = 34 III = 16 IV = 0	23.38 (2.5)	50	randomized; whole head	6 months	138.4 (38.03)	2.3 (24.20)
Albon 2016 [16]	Oral Marine Complex	II to III	42.8 (7.7)	30	randomized; whole head	Day 180	159.7 (46.26)	12.69 (12.11)
	Placebo	II to III	46.1 (7.6)	30	randomized; whole head	Day 180	150 (42.14)	−5.29 (12.10)
Mostafa 2021 [17]	Topical Cetirizine 1%	I to II = 6 III to IV = 5	34.5 (7)	18	randomized; whole head	24 weeks	102.3 (29,1)	22.7 (18.38)
	Topical Minoxidil 5%	I to II = 12 III to IV = 7	33.2 (8.4)	12	randomized; whole head	24 weeks	113 (27.4)	30.0 (18.79)
Hashimoto 2022 [18]	Topical Watercress 2%	III to VI	20–55 years	19	randomized; whole head	6 months	change presented in graph	12.8 (27.96)
	Placebo	III to VI	20–55 years	18	randomized; whole head	6 months	change presented in graph	−0.13 (31.75)
Amiri 2023 [19]	Topical Diamino-pyrimidine oxide 1%	I = 1 II = 1 III = 3 IV = 5 V = 1 VI = 2 VII = 1	30.5 (2.9)	14	randomized; whole head	24 weeks	only mean change reported	19.5 (4.86)
	Topical Minoxidil 2%	I = 1 II = 4 III = 3 IV = 3 V = 2 VI = 1 VII = 1	28.2 (3.1)	15	randomized; whole head	24 weeks	only mean change reported	11.2 (1.94)
Fischer 2012 [20]	Topical Melatonin	I to II	18–41 years	35	single arm	6 months	123 (15.39)	50.41 (35.58)
Baldari 2007 [21]	Topical Melatonin	II = 16 III = 8 III-v = 7	27 (20–39 years)	29 (16 patients received treatment in the parietal scalp region; 15 patients received treatment in the frontal scalp region)	single arm	Day 180	Parietal: 142.13 (45.94) Frontal: 153.47 (56.89)	36.9 (24.26) ^D^
Wessagowit 2016 [22]	Topical Saw Palmetto	III = 22% III-a = 6% III-v = 10% IV = 26% IV-a = 6% V = 18% V-a = 2% VI = 10%	35.12 (6.25)	49	single arm	24 weeks	mean per 2.54 cm^2^ (95% CI): 1736.369 (1666.325, 1806.341)	33.60 (74.17)
Amini 2025 [23]	Exosome formulation (subcutaneous injection)	II to III	20–50 years	10	randomized; whole head	16 weeks	only mean change reported	33.12 (20.93)
	Placebo	II to III	20–50 years	10	randomized; whole head	16 weeks	only mean change reported	7.33 (21.34)
Singh 2020 [24] ^E^	Topical Minoxidil 5%	II to V-a ^F^	26.9 (3.9)	17	randomized; whole head	6 months	90.65 (41.39)	33.3 (54.47)
	Placebo	II to V-a ^F^	25.6 (3.2)	19	randomized; whole head	6 months	90.05 (39.02)	−1.05 (54.04)
Sakr 2013 [25] ^E^	Topical Minoxidil 5%	II to IV	25–30 years	11	randomized; whole head	24 weeks	122.3,14.9	18.55 (8.90)
	Placebo	II to IV	25–30 years	10	randomized; whole head	24 weeks	170.5, 11.9	−19.90 (14.50)
Civatte 1987 [26] ^G^	Topical Minoxidil 2%	III-v = 43 IV = 35 V = 47	33.1 (18–49)	125	randomized; whole head	24 weeks	information presented graphically	33.39 (17.59)
	Placebo	III-v = 44 IV = 40 V = 38	33.1 (18–49)	122	randomized; whole head	24 weeks	information presented graphically	28.18 (21.83)
Petzoldt 1988 [27]	Topical Minoxidil 2%	III-v or IV	32.9 (17–49)	101	randomized; whole head	24 weeks	(per 2.5 cm diameter (SEM)) 201.6 (14.4)	16.17 (19.07)
	Placebo	III-v or IV	32.9 (17–49)	100	randomized; whole head	24 weeks	(per 2.5 cm diameter (SEM)) 201.4 (13)	6.1 (14.96)
Anderson 1988 [28]	Topical Minoxidil 2%	III-v or IV	32.8 (19–49)	75	randomized; whole head	24 weeks	(per 2.5 cm diameter (SEM)) 311.6 (16)	25.35 (22.32)
	Placebo	III-v or IV	32.8 (19–49)	76	randomized; whole head	24 weeks	(per 2.5 cm diameter (SEM)) 324 (16.7)	10.2 (18.85)
Dutr ée -Meulenberg 1988 [29]	Topical Minoxidil 2%	III-v or IV	34.3 (19–49)	74	randomized; whole head	24 weeks	(per 2.5 cm diameter (SEM)) 415.3 (19.6)	17.49 (21.32)
	Placebo	III-v or IV	34.3 (19–49)	70	randomized; whole head	24 weeks	(per 2.5 cm diameter (SEM)) 440.7 (20.4)	5.9 (21.67)
Olsen 1986 [30] ^E^	Topical Minoxidil 2%	III-v = 12 IV = 7	35.4 (4)	19	randomized; whole head	6 months	528 (116)	35 (19.8)
	Placebo	III-v = 13 IV = 6	37 (6.3)	19	randomized; whole head	6 months	527 (104)	18 (17.8)
Rushton 1989 [31]	Topical Minoxidil 2%	III to VI	18–49 years	11	randomized; whole head	6 months	information presented graphically	10.3 (47.63)
	Placebo	III to VI	18–49 years	6	randomized; whole head	6 months	information presented graphically	−4.64 (56.26)

AGA, androgenetic alopecia; SD, standard deviation; SEM, standard error of the mean. ^A^ Wherever mean age is not provided, the range was reported. ^B^ Where not reported, values were calculated from available data. ^C^ Hair loss severity was assessed using the Ogata scale. ^D^ Hair density change is reported only for the subgroup of patients showing improvement. ^E^ Only treatment groups relevant to the network meta-analysis are included; additional study arms were excluded. ^F^ AGA severity was reported for the entire study population. ^G^ Patient characteristics reflect all enrolled participants; 22 patients were lost to follow-up.

**Table 3:** Overview of mechanisms of action of off-label treatments of male AGA.

Therapy	Mechanism of Action
Topical Finasteride	(1) 5α-reductase inhibitor [33]
Botulinum Toxin	(1) Reduce scalp muscle tension (2) Improve microcirculation (3) Reduce inflammation [34]
Procyanidin	(1) Antioxidant (2) Anti-inflammatory (3) Protect dermal papilla cells from TGF-β-induced apoptosis [13,35]
Rosemary Oil	(1) Antioxidant (2) Anti-inflammatory (3) Antimicrobial [33]
Marine Complex	(1) Antioxidant (2) Stimulate dermal papilla proliferation (3) Increase alkaline phosphatase (hair growth indicator) [36]
Cetirizine	(1) Lower PGD_2_ (hair growth inhibitor) (2) Increase PGE_2_ (hair growth promoter) [17]
Watercress Extract	(1) Upregulate R-spondin1 (hair growth promoter) (2) Inhibit DKK1 (hair growth inhibitor) [18]
Diaminopyrimidine Oxide	(1) Stimulate growth factor expression (2) Promote follicular cycling [37]
Melatonin	(1) Antioxidant (2) Anti-inflammatory (3) Anti-androgen [20]
Saw Palmetto	(1) 5α-reductase inhibitor (type I & II) (2) Increase conversion of DHT to weaker metabolite (androstenediol) [2,33]
Exosomes	(1) Stimulate dermal papilla cell activity (2) Promote follicular cycling (3) Counteract DHT-mediated inhibition [38]

## Data Availability

No new primary data were created or analyzed in this study.

## Supplementary Materials

The following are available online at https://www.mdpi.com/article/10.3390/medicina62071282/s1, Table S1: Details of search queries. Figure S1: Qualitative summary of the study-level evaluation of risk of bias is presented in the ‘traffic plot’. Table S2: Calculation guide. Table S3: Kilim plot—description and explanation. Figure S2: League table of pairwise relative effects for interventions in male AGA. Table S4: Results of Node-splitting analysis of inconsistency for base model and sensitivity analyses; (A): Results of Node-splitting analysis of inconsistency (base model); (B): Results of Node-splitting analysis of inconsistency (sensitivity analysis: RCT only—fixed effects); (C): Results of Node-splitting analysis of inconsistency (sensitivity analysis: RCT only—random effects); (D): Results of Node-splitting analysis of inconsistency (sensitivity analysis: RCT only and without Amini et al. 2025—fixed effects); (E): Results of Node-splitting analysis of inconsistency (sensitivity analysis: RCT only and without Amini et al. 2025—random effects). Table S5: Model diagnostics.

## Abbreviations

The following abbreviations are used in this manuscript:

AGA	Androgenetic alopecia
BTA	Botulinum toxin A
CrI	Credible interval
DHT	Dihydrotestosterone
DKK1	Dickkopf-1
DPO	Diaminopyrimidine oxide
FDA	U.S. Food and Drug Administration
HGF	Hepatocyte growth factor
MAPK/MEK	Mitogen-activated protein kinase
MD	Mean difference
N	Number of patients
NMA	Network Meta-Analysis
NR	Not reported
OSF	Open Science Framework
PGD_2_	Prostaglandin D_2_
PGE_2_	Prostaglandin E_2_
PICO	Patient, intervention, comparator, and outcome
PRISMA	Preferred Reporting Items for Systematic Reviews and Meta-Analyses
RCT	Randomized controlled trial
RE	Relative effects
RSPO1	R-spondin 1
SD	Standard deviation
SE	Standard error
SUCRA	Surface under the cumulative ranking curve
TGF-β	Transforming growth factor-beta
VEGF	Vascular endothelial growth factor
