# Supplementary material for: Beyond Minoxidil: Off-Label Therapies for Male Androgenetic Alopecia—A Systematic Review with Network Meta-Analyses
Source: Medicina (Kaunas). 2026 Jul 3;62(7):1282. doi: 10.3390/medicina62071282 (PMC13414076; doi:10.3390/medicina62071282)
Supplement: Supplementary file 1 [file medicina-62-01282-s001.zip › medicina-4336467-supplementary.pdf]

Beyond Minoxidil: Off-Label Therapies for Male Androgenetic Alopecia—A Systematic Review with Network Meta-Analyses

Aditya K. Gupta [1,2], Shannon A.H. Compton [2], Amanda Liddy [2], Mesbah Talukder [2,3], Tong Wang [2], and Mary A. Bamimore [2]

[1] Division of Dermatology, Temerty Faculty of Medicine, University of Toronto, Toronto, ON M5S 1A8, Canada
[2] Mediprobe Research Inc., London, ON N5X 2P1, Canada
[3] SSchool of Pharmacy, BRAC University, Dhaka 1212, Bangladesh
* Correspondence: agupta@mediproberesearch.com or aditya.gupta@utoronto.ca

Table S1: Details of search queries 2
Figure S1: Qualitative summary of the study-level evaluation of risk of bias is presented in the 'traffic plot' 3
Table S2: Calculation guide 4
Table S3: Kilim plot—description and explanation 5
Figure S2: League table of pairwise relative effects for interventions in male AGA. 6
Table S4. Results of Node-splitting analysis of inconsistency for base model and sensitivity analyses 7
Table S4A. Results of Node-splitting analysis of inconsistency (base model) 7
Table S4B. Results of Node-splitting analysis of inconsistency (sensitivity analysis: RCT only—fixed effects) 8
Table S4C. Results of Node-splitting analysis of inconsistency (sensitivity analysis: RCT only—random effects) 9
Table S4D. Results of Node-splitting analysis of inconsistency (sensitivity analysis: RCT only and without Amini et al 2025—fixed effects) 10
Table S4E. Results of Node-splitting analysis of inconsistency (sensitivity analysis: RCT only and without Amini et al 2025—random effects) 11
Table S5: Model diagnostics 12

Table S1: Details of search queries

Search Queries Used Across Electronic Databases

Database	Search details	Notes
Web of Science	"androgenetic alopecia" OR "pattern baldness" OR "pattern hair loss" (Abstract) and "efficacy" OR "effectiveness" OR "impact" OR "trial" OR effect (Abstract)	For each database, there was no date restriction, and only evidence in English language were included
Scopus	(ABS ("Androgenetic alopecia" OR "pattern hair loss" OR "pattern baldness") AND ABS ("efficacy" OR "effectiveness" OR "trial" OR "impact"))	
ClinicalTrials.gov	"androgenetic Alopecia" OR "pattern hair loss" OR "pattern baldness" | Completed studies | Male participants | Interventional studies | Studies with results	

Figure S1: Qualitative summary of the study-level evaluation of risk of bias is presented in the ‘traffic plot’

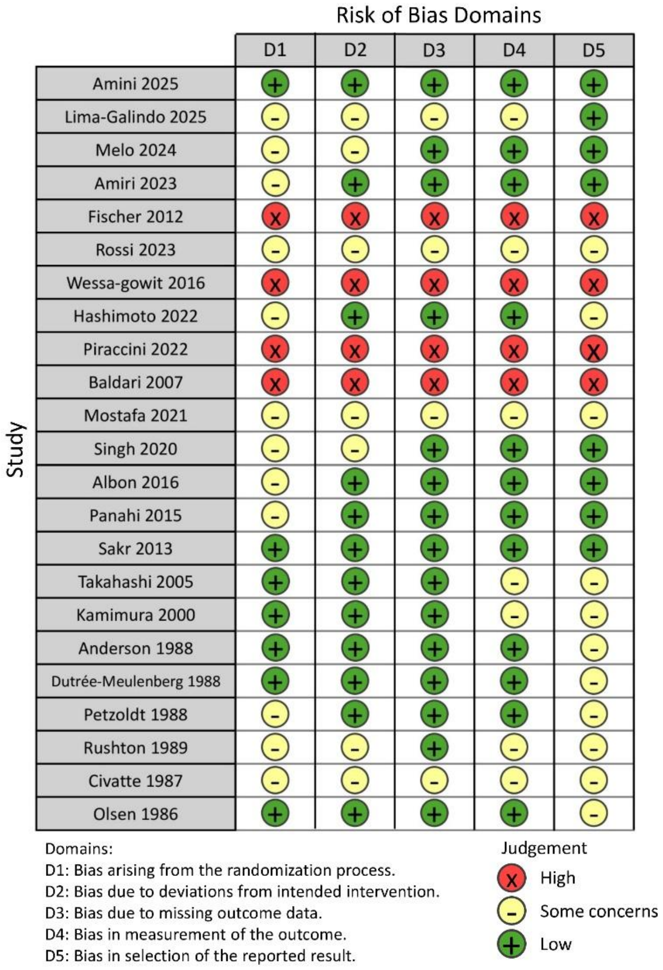

Table S2: Calculation guide

- When hair counts were provided, we estimated hair density (in hairs/cm^2) by dividing the value of hair counts by the target area used. For instance, for Wessagowit et al. (2016) we divided baseline and 24-week hair counts by the target area (i.e., 2.54 cm^2).
- When the baseline and final (i.e., follow-up) hair density values were provided, we did the following to obtain the mean and ±standard deviation (SD) for the 6-month change in hair density (hairs/cm^2)
    - Mean = follow up — baseline (i.e., follow up value minus baseline value)
    - ±Standard deviation[Ͳ] = $\mathrm{SD}_{E,\mathrm{change}} = \sqrt{\mathrm{SD}^2_{E,\mathrm{baseline}} + \mathrm{SD}^2_{E,\mathrm{final}} - (2 \times \mathrm{Corr} \times \mathrm{SD}_{E,\mathrm{baseline}} \times \mathrm{SD}_{E,\mathrm{final}})}$

  Corr = 0.8
- When values were presented graphically, we used WebPlotDigitizer[‡] to extract hair values
- When 95% confidence intervals (CI) were provided, we used the following formulae[Ͳ]:
    - Standard error = $(\text{upper limit} - \text{lower limit})/3.92$
    - Standard deviation = Standard error * square root of sample size

- When medians and interquartile ranges were provided we used the following paper as a guide:
  Hozo, S.P., Djulbegovic, B. & Hozo, I. Estimating the mean and variance from the median, range, and the size of a sample. BMC Med Res Methodol 5, 13 (2005). https://doi.org/10.1186/1471-2288-5-13

More detail is available under the Cochrane Collaboration guides[Ͳ]

References:

[Ͳ]*Higgins JP, Li T, Deeks JJ (editors). Chapter 6: Choosing effect measures and computing estimates of effect [last updated August 2023]. In: Higgins JP, Thomas J, Chandler J, Cumpston M, Li T, Page MJ, et al, editor(s). Cochrane Handbook for Systematic Reviews of Interventions version 6.5. Cochrane, 2024. Available from cochrane.org/handbook.https://www.cochrane.org/authors/handbooks-and-manuals/handbook/current/chapter-06*

[‡]*Drevon, D. D., Fursa, S. R., & Malcolm, A. L. (2017). Intercoder Reliability and Validity of WebPlotDigitizer in Extracting Graphed Data. Behavior Modification, 41(2), 323–339. DOI: 10.1177/0145445516673998*

Table S3: Kilim plot—description and explanation

To facilitate comparison of treatment rankings across the primary (i.e., base) and sensitivity analyses, SUCRA values were visualized using a kilim plot, a graphical approach developed by Seo et al. (2021) for the visualization of results from multiple network meta-analyses. In this plot, treatments are displayed on the y-axis (i.e., vertical axis) and network meta-analysis models on the x-axis (i.e., horizontal axis); SUCRA values are represented using a colour gradient ranging from 0 to 1 (or 0% to 100%). Similar colour gradient across columns indicate similarity in treatment rankings between models. The kilim plot therefore provides an intuitive visual assessment of the consistency of treatment rankings.

Reference:

Seo M, Furukawa TA, Veroniki AA, Pillinger T, Tomlinson A, Salanti G, Cipriani A, Efthimiou O. The Kilim plot: A tool for visualizing network meta-analysis results for multiple outcomes. Research Synthesis Methods. 2021;12(1):86–95.

Figure S2: League table of pairwise relative effects for interventions in male AGA.
Each cell represents mean difference and corresponding 95% credible interval.

MNX 5% (Topical)															
2.52 (-8.04, 13.37)	MEL (Topical)														
8.41 (-0.19, 16.94)	5.89 (-5.86, 17.66)	DPO 1% (Topical)													
8.84 (-5.04, 22.92)	6.32 (-9.91, 22.72)	0.43 (-12.02, 12.54)	PC 1% (Topical)												
10.64 (-5.42, 27.38)	8.12 (-10.06, 25.86)	2.23 (-12, 17.35)	1.8 (-16.53, 19.78)	SP (Topical)											
11.99 (-3.27, 27.03)	9.47 (-7.87, 26.62)	3.58 (-10.03, 18.02)	3.15 (-14.53, 20.92)	1.35 (-18.45, 21.14)	EXO (SC)										
**13.83 (4.05, 23.64)**	11.31 (-0.57, 23.34)	5.42 (-5.62, 16.34)	4.99 (-10.51, 21.38)	3.19 (-14.78, 20.31)	1.84 (-14.92, 19.03)	CETI 1% (Topical)									
**14.86 (4.83, 24.83)**	**12.33 (0.07, 24.62)**	6.44 (-0.67, 13.62)	6.02 (-7.32, 19.54)	4.22 (-11.59, 20.19)	2.87 (-12.23, 17.75)	1.03 (-11.44, 12.75)	FIN (Topical)								
**15.17 (2.87, 27.39)**	12.64 (-1.69, 26.99)	6.75 (-2.84, 16.68)	6.33 (-8.09, 21.69)	4.53 (-12.92, 21.71)	3.18 (-13.06, 19.52)	1.34 (-12.74, 15.54)	0.31 (-11.18, 11.99)	ROS (Topical)							
**15.44 (2.23, 28.52)**	12.92 (-2.4, 28.2)	7.03 (-4.11, 17.93)	6.6 (-9.02, 22.16)	4.8 (-13.12, 22.59)	3.45 (-13.59, 20.63)	1.61 (-13.25, 16.56)	0.58 (-11.72, 12.83)	0.27 (-13.86, 14.14)	PC 0.7% (Topical)						
**15.81 (5.94, 25.51)**	**13.28 (0.9, 25.91)**	**7.39 (0.7, 14.21)**	6.96 (-6.17, 20.5)	5.16 (-10.94, 21.12)	3.81 (-10.8, 18.39)	1.97 (-10.35, 13.7)	0.95 (-7.56, 9.42)	0.64 (-10.29, 12.14)	0.36 (-11.61, 12.18)	MC (Oral)					
**16.34 (8.01, 24.38)**	**13.82 (2.55, 25.32)**	**7.93 (5.2, 10.67)**	7.5 (-4.66, 19.63)	5.7 (-9.26, 19.86)	4.35 (-9.81, 18.04)	2.51 (-8.22, 13.24)	1.48 (-5.43, 8.53)	1.17 (-8.15, 10.59)	0.9 (-9.68, 11.58)	0.54 (-5.99, 6.86)	MNX 2% (Topical)				
**18.29 (3.12, 33.4)**	15.77 (-1.23, 32.7)	9.88 (-3.04, 23.7)	9.45 (-7.77, 26.84)	7.65 (-12.03, 26.92)	6.3 (-12.33, 25.12)	4.46 (-12.58, 21.44)	3.43 (-10.83, 18.55)	3.12 (-12.69, 19.36)	2.85 (-13.61, 19.64)	2.49 (-11.15, 16.6)	1.95 (-10.95, 15.44)	WC 2% (Topical)			
**22.17 (1.8, 42.25)**	19.65 (-2.35, 40.57)	13.76 (-5.58, 32.45)	13.33 (-9.38, 35.57)	11.53 (-12.69, 35.82)	10.18 (-13.02, 32.13)	8.34 (-13.77, 29.92)	7.31 (-12.93, 27.08)	7 (-13.37, 27.47)	6.73 (-14.49, 28.48)	6.37 (-13.16, 25.6)	5.83 (-12.99, 24.58)	3.88 (-18.77, 26.2)	BTX (SC)		
**25.44 (17.62 33.01)**	**22.92 (12.02, 34.04)**	**17.03 (13.34, 20.75)**	**16.6 (4.94, 28.53)**	**14.8 (0.03, 28.95)**	13.45 (-0.31, 26.91)	**11.61 (0.99 , 21.87)**	**10.59 (4.27, 17.09)**	**10.28 (0.75, 19.86)**	10 (-0.2, 20.26)	**9.64 (3.79, 15.44)**	**9.1 (6.49, 11.72)**	7.15 (-5.72, 19.93)	3.27 (-15.53, 22.17)	Control	
**29.03 (17.75, 40.66)**	**26.5 (12.75, 40.51)**	**20.61 (11.67, 29.81)**	**20.19 (5.49, 34.62)**	**18.38 (1.27, 34.5)**	**17.04 (0.5, 33.06)**	**15.19 (2.02, 28.84)**	**14.17 (3.63, 25.04)**	**13.86 (1.25, 26.74)**	**13.58 (0.22, 26.54)**	**13.22 (2.85, 23.55)**	**12.69 (3.92, 21.65)**	10.74 (-4.65, 25.86)	6.85 (-13.26, 26.6)	3.58 (-4.8, 12.21)	BTX (IM + SC)

U.S. FDA Approved

p <0.05

BTX, botulinum toxin
CETI, cetirizine
DPO, Diaminopyrimidine oxide
EXO, exosome
FIN, finasteride
IM, intramuscular
MC, marine complex
MEL, melatonin
MX, minoxidil
PC, Procyanidin
ROS, rosemary
SC, subcutaneous
SP, Saw palmetto
WC, watercress

Table S4. Results of Node-splitting analysis of inconsistency for base model and sensitivity analyses

Table S4A. Results of Node-splitting analysis of inconsistency (base model)

Comparison	p-value	Credible interval
Minoxidil 5% topical vs. Placebo	0.762900	
Direct		-38. (-51., -25.)
Indirect		-43. (-76., -11.)
network		-39. (-50., -27.)
Minoxidil 5% topical vs. Finasteride topical	0.575500	
Direct		-35. (-73., 2.0)
Indirect		-24. (-42., -4.4)
network		-25. (-40., -10.)
Placebo vs Finasteride topical	0.800275	
Direct		14. (1.2, 27.)
Indirect		10. (-24., 42.)
network		14. (2.8, 25.)
Finasteride topical vs. Melatonin topical	0.957400	
Direct		31. (3.3, 60.)
Indirect		30. (4.0, 57.)
network		30. (12., 48.)

Table S4B. Results of Node-splitting analysis of inconsistency (sensitivity analysis: RCT only—fixed effects)

Comparison	p-value	Credible interval
Placebo vs. Finasteride topical	0.601	
Direct		14. (0.97, 28.)
Indirect		4.1 (-35., 45.)
network		14. (1.2, 25.)
Placebo vs. Minoxidil 5% topical	0.580	
Direct		38. (24., 52.)
Indirect		50. (8.6, 90.)
network		39. (27., 51.)
Finasteride topical vs. Minoxidil 5% topical	0.557	
Direct		35. (-2.4, 72.)
Indirect		23. (3.8, 43.)
network		25. (9.8, 42.)

Table S4C. Results of Node-splitting analysis of inconsistency (sensitivity analysis: RCT only—random effects)

Comparison	p-value	Credible interval
Placebo vs. Finasteride topical	0.603	
Direct		14. (0.72, 28.)
Indirect		3.2 (-37., 44.)
network		14. (1.5, 25.)
Placebo vs. Minoxidil 5% topical	0.628	
Direct		38. (24., 51.)
Indirect		48. (7.6, 88.)
network		39. (27., 52.)
Finasteride topical vs. Minoxidil 5% topical	0.582	
Direct		36. (-4.9, 73.)
Indirect		23. (4., 43.)
network		26. (9.7, 42.)

Table S4D. Results of Node-splitting analysis of inconsistency (sensitivity analysis: RCT only and without Amini et al 2025—fixed effects)

Comparison	p-value	Credible interval
Finasteride topical vs. Minoxidil 5% topical	0.595	
Direct		35. (-3.2, 71.)
Indirect		24. (3.8, 42.)
network		26. (9.2, 42.)
Finasteride topical vs. Placebo	0.560	
Direct		-14. (-28., -1.3)
Indirect		-2.5 (-43., 37.)
network		-14. (-25., -0.81)
Minoxidil 5% topical vs. Placebo	0.582	
Direct		-38. (-51., -24.)
Indirect		-49. (-89., -8.6)
network		-39. (-52., -26.)

Table S4E. Results of Node-splitting analysis of inconsistency (sensitivity analysis: RCT only and without Amini et al 2025—random effects)

Comparison	p-value	Credible interval
Finasteride topical vs. Minoxidil 5% topical	0.594	
Direct		35. (-4.7, 72.)
Indirect		23. (3.8, 43.)
network		26. (9.4, 43.)
Finasteride topical vs. Placebo	0.614	
Direct		-14. (-28., -0.90)
Indirect		-3.9 (-44., 38.)
network		-14. (-25., -0.52)
Minoxidil 5% topical vs. Placebo	0.582	
Direct		-38. (-51., -24.)
Indirect		-50. (-90., -8.8)
network		-39. (-52., -26.)

Table S5: Model diagnostics

Measure	Base NMA	RCT-only (fixed effects)	RCT-only (random-effects)	RCT-only excluding Amini et al. (2025) (fixed effects)	RCT-only excluding Amini et al. (2025) (random effects)
Deviance Information Criterion (DIC)	88	77.6	75.9	74.2	70.5
Tau (Posterior mean between-study heterogeneity)	—	—	5.95 (95% CI: 0.96 – 11.54)	—	5.64 (95% CI: 2.74–11.46)

- Model fit statistics—the deviance information criterion (DIC);
- Heterogeneity parameters—Tau (τ) for the random-effects models.
